# The Relationship Between Default Mode and Dorsal Attention Networks Is Associated With Depressive Disorder Diagnosis and the Strength of Memory Representations Acquired Prior to the Resting State Scan

**DOI:** 10.3389/fnhum.2022.749767

**Published:** 2022-02-21

**Authors:** Skye Satz, Yaroslav O. Halchenko, Rachel Ragozzino, Mora M. Lucero, Mary L. Phillips, Holly A. Swartz, Anna Manelis

**Affiliations:** ^1^Department of Psychiatry, Western Psychiatric Institute and Clinic, University of Pittsburgh Medical Center, University of Pittsburgh, Pittsburgh, PA, United States; ^2^Department of Psychological and Brain Sciences, Dartmouth College, Hanover, NH, United States

**Keywords:** resting state, fMRI, functional connectivity, depression, memory, default mode network, dorsal attention network

## Abstract

Previous research indicates that individuals with depressive disorders (DD) have aberrant resting state functional connectivity and may experience memory dysfunction. While resting state functional connectivity may be affected by experiences preceding the resting state scan, little is known about this relationship in individuals with DD. Our study examined this question in the context of object memory. 52 individuals with DD and 45 healthy controls (HC) completed clinical interviews, and a memory encoding task followed by a forced-choice recognition test. A 5-min resting state fMRI scan was administered immediately after the forced-choice task. Resting state networks were identified using group Independent Component Analysis across all participants. A network modeling analysis conducted on 22 networks using FSLNets examined the interaction effect of diagnostic status and memory accuracy on the between-network connectivity. We found that this interaction significantly affected the relationship between the network comprised of the medial prefrontal cortex, posterior cingulate cortex, and hippocampal formation and the network comprised of the inferior temporal, parietal, and prefrontal cortices. A stronger positive correlation between these two networks was observed in individuals with DD who showed higher memory accuracy, while a stronger negative correlation (i.e., anticorrelation) was observed in individuals with DD who showed lower memory accuracy prior to resting state. No such effect was observed for HC. The former network cross-correlated with the default mode network (DMN), and the latter cross-correlated with the dorsal attention network (DAN). Considering that the DMN and DAN typically anticorrelate, we hypothesize that our findings indicate aberrant reactivation and consolidation processes that occur after the task is completed. Such aberrant processes may lead to continuous “replay” of previously learned, but currently irrelevant, information and underlie rumination in depression.

## Introduction

Resting state neuroimaging techniques examine brain activation and functional connectivity in the absence of stimuli or tasks. Previous studies characterized a set of networks in which brain regions co-activate during resting state (Beckmann et al., 2005; Damoiseaux et al., 2006; De Luca et al., 2006; Smith et al., 2009; Biswal et al., 2010; van den Heuvel and Hulshoff Pol, 2010; Yeo et al., 2011; Lv et al., 2018). Resting state functional connectivity may be related to sustained information processing (Gusnard and Raichle, 2001) as well as environment monitoring and internal thought processes (Buckner et al., 2008) that can dynamically change based on the experiences preceding the resting state scan (Daselaar et al., 2010; Sami et al., 2014; Cecchetto et al., 2019) as well as predict task performance that follows the resting state scan (Tambini et al., 2010; Sala-Llonch et al., 2012; López Zunini et al., 2013; Reineberg et al., 2015).

Resting state functional connectivity is altered in a variety of psychiatric illnesses (Greicius, 2008; Zhang and Raichle, 2010) including depressive disorders (Greicius et al., 2007; Woodward and Cascio, 2015; Zhang et al., 2021). Depressive disorders (DD) (e.g., major depressive and persistent depressive disorders) are characterized by feelings of sadness, loss of interest and motivation, fatigue, anhedonia, feelings of hopelessness, worthlessness and guilt, as well as changes in sleeping, eating, and overall cognitive and psychosocial functioning (First et al., 2015). In addition, individuals with DD may experience memory dysfunction (Burt et al., 1995; Williams et al., 2007; Hamilton and Gotlib, 2008; Vasic et al., 2009; Gotlib and Joormann, 2010; Dillon and Pizzagalli, 2018; Ge et al., 2019). Previous neuroimaging studies found elevated connectivity within the default mode network (DMN) as well as between the DMN and non-DMN regions, including the dorsal attention network (DAN), insula, thalamus, and subgenual cingulate in individuals with DD relative to healthy controls (HC) (Sheline et al., 2010; Scalabrini et al., 2020). Clinical features of depression, such as symptom severity, illness duration, medication status and, most notably, ruminative and self-referential thought, have been linked to disruption of the cognitive control, salience, and default mode networks (Greicius et al., 2007; Bluhm et al., 2009; Sheline et al., 2010; Zeng et al., 2012; Brakowski et al., 2017; Scalabrini et al., 2020).

Mind wandering (Kucyi and Davis, 2014; Chou et al., 2017) and experiencing self-referential thoughts (Sheline et al., 2009) and spontaneous rumination (Rosenbaum et al., 2017) might be related to intentional or unintentional reactivation of memory representations that participants encountered prior to the resting state scan and memory consolidation (Sami et al., 2014). During memory consolidation, recently formed memories change into stable memory representations through reorganization and transitioning of hippocampal-dependent to neocortex supported memories (Squire et al., 2015). Memory representations can be reactivated during stimulus re-study (Vilberg and Davachi, 2013), memory tests (Manelis et al., 2011), or spontaneously during rest following stimuli encoding (Deuker et al., 2013; Wittkuhn and Schuck, 2021). The latter is particularly relevant for the study of psychiatric disorders as it might explain such phenomena as depressive rumination, obsessive thoughts, and other aberrant forms of cognition. Given that the majority of previous studies focused on healthy individuals and administered the resting state scan following stimulus encoding and prior to a memory test (Tambini et al., 2010; Tambini and Davachi, 2013; Schlichting and Prestona, 2014; Tompary et al., 2015; Murty et al., 2017), little is known about how the accuracy of memories acquired prior to the resting state scan is related to functional connectivity within and between resting state networks in individuals with DD, compared to HC.

Our study examined whether the relationship between memory retrieval accuracy for recently encoded stimuli and resting state functional connectivity was moderated by diagnostic status. The experimental paradigm consisted of the encoding phase, during which participants encountered pictures of objects and tried to memorize them, the forced-choice recognition task, and the subsequent resting state scan in which the memorized stimuli were no longer relevant. Memory encoding, retrieval, and consolidation involve the hippocampus (Nadel and Moscovitch, 1997; Squire et al., 2015). Considering that the hippocampus is a part of the DMN (Buckner et al., 2008; Andrews-Hanna et al., 2010b) that has aberrant functional connectivity in individuals with DD (Sheline et al., 2009; Marchetti et al., 2012; Scalabrini et al., 2020) and that individuals with DD have difficulty disengaging from previously learned information (Xia and Evans, 2020), we hypothesized that memory accuracy in individuals with DD would be associated with aberrant DMN connectivity relative to HC.

## Materials and Methods

### Participants

This study was approved by the University of Pittsburgh Institutional Review Board. Participants were recruited from the community, universities, and counseling and medical centers through referrals and advertisements. Written informed consent was obtained from all participants. Participants were right-handed, fluent in English, and matched on age, sex, and IQ. HC had no personal or family history of psychiatric disorders. Symptomatic participants met DSM-5 criteria for a depressive disorder (DD) such as major depressive disorder (MDD) or persistent depressive disorder (PDD).

Neuroimaging data were collected from 114 participants (53 HC and 61 DD). A total of 17 participants were excluded from the analyses due to excessive motion (>2 mm translation; 7 HC and 5 DD), poor data quality due to scanner noise (1 DD) or falling asleep during the resting state scan (1 HC and 3 DD). The final sample included 45 HC and 52 DD for a total of 97 participants. Of the 52 individuals with DD, 35 were diagnosed with MDD and 17 were diagnosed with PDD. MDD and PDD differ in some characteristics, such as symptom duration and severity, yet display substantial overlap in symptomatology (First et al., 2015). Of the 17 individuals with PDD, 13 had intermittent major depressive episodes, a clinical feature making the symptom profiles of MDD and PDD difficult to differentiate (Schramm et al., 2020).

### Clinical Assessment

All diagnoses were made by a trained clinician and confirmed by a psychiatrist according to DSM-5 criteria using the SCID-5 clinical interview (First et al., 2015). We also collected information about current depression symptoms using the Hamilton Depression Rating Scale (HDRS-25) (Hamilton, 1960) and lifetime dimensional symptoms of depression using the MOODS-SR (Dell’Osso et al., 2002). A total psychotropic medication load was calculated for each participant, with greater numbers and doses of medications corresponding to a greater medication load (Hassel et al., 2008; Manelis et al., 2016). Exclusion criteria included a history of head injury, metal in the body, pregnancy, claustrophobia, neurodevelopmental disorders, systemic medical illness, premorbid IQ < 85 per the National Adult Reading Test (Nelson, 1982), current alcohol/drug abuse, the YMRS (Young et al., 1978) scores >10 at scan, and meeting criteria for any psychotic-spectrum disorder. Table 1 reports group statistics for participants’ demographic and clinical characteristics.

**TABLE 1:** Demographic and clinical characteristics.

	HC	UD	Statistics HC vs. UD
Number of participants	45	52	
Gender composition (number females)	33	42	chi^2^ = 0.40, *p* = 0.53
UD diagnoses (MDD/PDD)	na	35/17	na
Age (years)	29.02 (1.00)	28.20 (0.93)	*t*(95) = 0.60, *p* = 0.55
BMI	26.12 (0.69)	25.25 (0.54)	*t*(95) = 1.01, *p* = 0.31
IQ (NART)	106.79 (0.81)	109.40 (1.01)	*t*(95) = −1.97, *p* = 0.052
Current depression severity (HDRS-25)	1.69 (0.31)	12.81 (0.98)	*t*(95) = −10.18, *p* < 0.001
Current mania severity (YMRS)	0.267 (0.12)	1.17 (0.20)	*t*(95) = −3.69, *p* < 0.001
Lifetime depression (MOODS-SR)	2.09 (0.34)	18.65 (0.57)	*t*(95) = −24.05, *p* < 0.001
Illness Onset (year of age)	na	14.90 (0.50)	na
Number of participants taking Antidepressants	na	34	na
Number of participants taking Mood stabilizers	na	2	na
Number of participants taking Antipsychotics	na	1	na
Number of participants taking Benzodiazepines	na	7	na
Number of participants taking Stimulants	na	4	na
A mean number of psychotropic medications	na	1.10 (0.14)	na
A mean total medication load	na	1.40 (0.19)	na
Number of participants with comorbid diagnoses	na	34	na

### Memory Encoding and Retrieval

**FIGURE 1:**
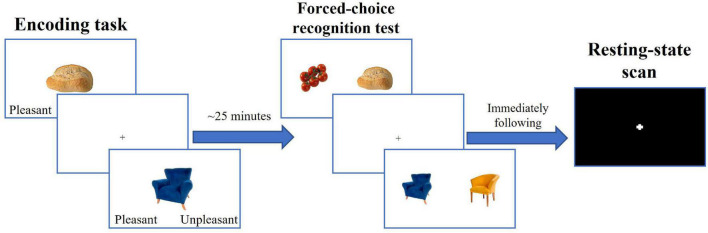
Experimental paradigm.

The experimental paradigm is depicted on Figure 1. During encoding, participants were presented with 48 pictures of common objects and food items (Blechert et al., 2014). To ensure deep information processing, they were instructed to rate each stimulus as pleasant or unpleasant (Richardson-Klavehn, 2010; Schott et al., 2013), and to memorize the presented stimuli. Approximately 25 min after encoding, participants performed the forced-choice object recognition task in which they were presented with pictures of an old (seen previously) and a novel (not seen previously) stimulus side-by-side and were required to select the old stimulus by pressing a corresponding button. The task consisted of 48 trials. The set of the novel stimuli was obtained from the same database as the set of the old stimuli (Blechert et al., 2014) and consisted of the items that categorically matched and visually resembled old stimuli (i.e., were from subordinate categories of old items). The pairings of novel and old stimuli were assigned randomly so that the old stimulus could be paired with a similar novel stimulus (e.g., blue chair and yellow chair), or a novel stimulus from a different category (e.g., bread and tomatoes). Each old stimulus had a closely resembling novel stimulus; therefore, participants had to have a detailed memory representation of an old stimulus to correctly identify it as old. The memory accuracy was calculated as a percent of correct responses relative to a total number of responses. While participants were scanned during both the encoding and forced-choice tasks, the neuroimaging results of these tasks will be described in separate reports.

The scans administered between encoding and forced choice object recognition tasks included spin-echo field maps in the posterior-to-anterior and anterior-to-posterior phase encoding directions (30 s each), a 7-min T1w anatomical scan, a 6.5-min T2w anatomical scan, and a forced-choice recognition task for face stimuli (approximately 10 min) that were encoded before the objects in the separate task.

### Resting State Scan

Immediately following the forced-choice recognition task, participants completed a 5-min resting state scan in which they were asked to look at the fixation star presented in the center of the screen and to stay awake. We used an eye-tracking camera system to monitor the participants’ wakefulness during the scan and made a note if a participant fell asleep or kept their eyes closed for more than 20 s. Participants also self-reported their wakefulness after the resting state scan.

### Neuroimaging Data Acquisition

The neuroimaging data were collected at the University of Pittsburgh/UPMC Magnetic Resonance Research Center (MRRC) using a 3T Siemens Prisma scanner with a 64-channel head coil. The acquisition series were named according to the ReproIn naming convention (Visconti di Oleggio Castello et al., 2020). The EPI data were collected in the anterior-to-posterior direction using a multi-band sequence (factor = 8, TR = 800 ms, resolution = 2 × 2 × 2 mm, FOV = 210 mm, TE = 30 ms, flip angle = 52°, 72 slices, 375 volumes). High-resolution T1w images were collected using the MPRAGE sequence (TR = 2,400 ms, resolution = 0.8 × 0.8 × 0.8 mm, 208 slices, FOV = 256 mm, TE = 2.22 ms, flip angle = 8°). Field maps were collected in the AP and PA directions using the spin echo sequence (TR = 8,000 ms, resolution = 2 × 2 × 2 mm, FOV = 210 mm, TE = 66 ms, flip angle = 90°, 72 slices).

### Data Analyses

#### Demographic and Clinical Data Analysis

The demographic and clinical characteristics were compared between DD and HC groups using *t*- and chi-square tests.

#### Memory Retrieval Analysis

We used *t*-tests to compare recognition accuracy and response time (RT) in DD vs. HC groups. RT was calculated across trials with correct responses only.

#### Neuroimaging Data Analysis

##### Preprocessing

The DICOM images were converted to BIDS standard using *heudiconv version 0.5.4* (Halchenko et al., 2019; RRID:SCR_017427) After that, the images were preprocessed using *fMRIPrep version 20.1.2* (Esteban et al., 2019; RRID:SCR_016216). First, a reference volume and its skull-stripped version were generated using a custom methodology of *fMRIPrep*. Head-motion parameters with respect to the BOLD reference (transformation matrices, and six corresponding rotation and translation parameters) are estimated before any spatiotemporal filtering using mcflirt (FSL 5.0.9, (Jenkinson et al., 2002; RRID:SCR_002823). BOLD runs were slice-time corrected using *3dTshift* (Cox and Hyde, 1997), (AFNI 20160207; RRID:SCR_005927). A B0-non-uniformity map (or fieldmap) was estimated based on two echo-planar imaging (EPI) references with opposing phase-encoding directions, with *3dQwarp* (Cox and Hyde, 1997) (AFNI 20160207). Based on the estimated susceptibility distortion, a corrected EPI (echo-planar imaging) reference was calculated for a more accurate co-registration with the anatomical reference. The BOLD reference was then co-registered to the T1w reference using *bbregister* (FreeSurfer) (Dale et al., 1999; RRID:SCR_001847) which implements boundary-based registration (Greve and Fischl, 2009). Co-registration was configured with six degrees of freedom. The BOLD time-series were resampled onto the following surfaces (FreeSurfer reconstruction nomenclature): *fsaverage*. The BOLD time-series (including slice-timing correction) were resampled onto their original, native space by applying a single, composite transform to correct for head-motion and susceptibility distortions. These resampled BOLD time-series will be referred to as preprocessed BOLD in original space, or just preprocessed BOLD. The BOLD time-series were resampled into standard space, generating a preprocessed BOLD run in MNI152NLin2009cAsym space. Automatic removal of motion artifacts using independent component analysis (ICA-AROMA) (Pruim et al., 2015) was performed on the preprocessed BOLD on MNI space time-series after removal of non-steady state volumes and spatial smoothing with an isotropic, Gaussian kernel of 6 mm full-width half-maximum (FWHM). Corresponding “non-aggressively” denoised runs were produced after such smoothing. Additionally, the aggressive noise-regressors were collected and placed in the corresponding confounds file. All resamplings were performed with a single interpolation step by composing all the pertinent transformations (i.e., head-motion transform matrices, susceptibility distortion correction when available, and co-registrations to anatomical and output spaces). Gridded (volumetric) resamplings were performed using *antsApplyTransforms* (ANTs), configured with Lanczos interpolation to minimize the smoothing effects of other kernels (Lanczos, 1964). Non-gridded (surface) resamplings were performed using mri_vol2surf (FreeSurfer).

**FIGURE 2:**
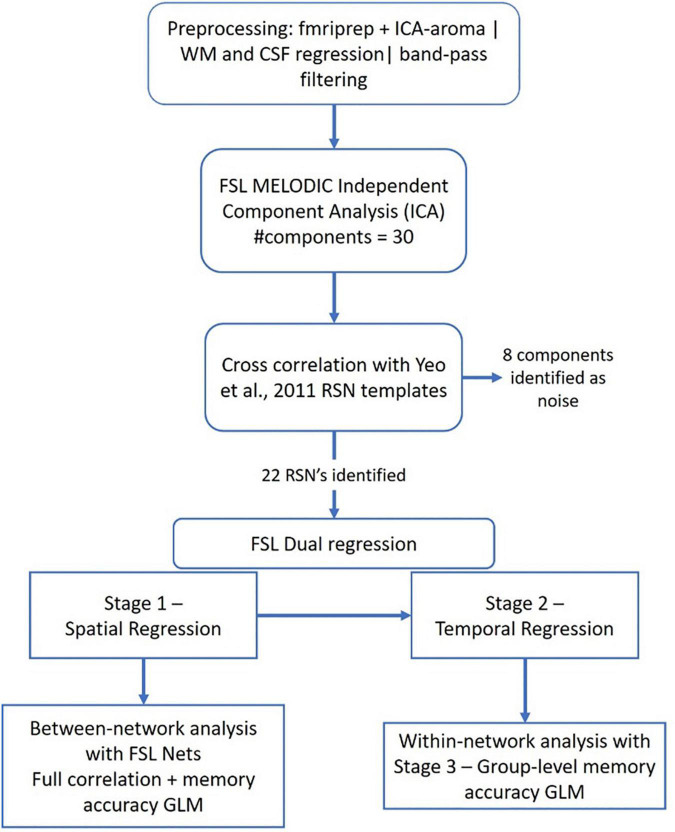
Processing pipeline for the resting state data analysis.

While *fMRIPrep* automatically extracts the three global signals within the CSF, the WM, and the whole-brain masks, they are extracted prior to removal of motion artifacts using ICA-AROMA (Pruim et al., 2015). Therefore, instead of using the automatically generated values, we extracted these signals after running the ICA-AROMA (from the files with the suffix “space-MNI152NLin6Asym_desc-smoothAROMAnonaggr_bold”) and regressed the CSF and WM from preprocessed resting state data. After that, the data were band-pass temporal filtered (0.01–0.1 Hz). The processing pipeline is depicted in Figure 2.

##### Group-Level Independent Component Analysis

The preprocessed, registered-to-MNI-space and band-pass filtered BOLD images described above served as input files to MELODIC group-ICA (Beckmann et al., 2005). We limited the number of components discovered by MELODIC to 30 to obtain large-scale networks. MELODIC uses a mixture model and the loss function to estimate the probability for a voxel to belong to the “active” or to the “background noise” classes. We implemented a default threshold of 0.5 that “places an equal loss on false positives and false negatives” (Beckmann and Smith, 2004) to obtain thresholded group-ICA components. Prior to performing any between-group statistical analyses, these thresholded group-ICA components (3D spatial maps) were cross-correlated with Yeo’s 7-network solution maps (Yeo et al., 2011) using *fslcc*, a tool that estimates spatial similarities between ICA outputs and/or other volumetric data. The group-ICA components whose cross-correlation values exceeded 0.2 were retained for the further analyses. The remaining components were visually examined for usability. The components that followed WM tracts, or resembled physiological noise were removed from the analyses.

##### Within- and Between-Network Analysis

All analyses were conducted on thresholded independent component maps. Within-network connectivity was assessed with a *dual regression* which generated both subject-specific component time courses and subject-specific spatial maps as output. Dual regression automatically utilizes *randomize* [with 5000 permutations and threshold free cluster enhancement (TFCE) to correct for multiple comparisons] for non-parametric permutation testing (Winkler et al., 2014) to perform a diagnostic status-by-memory accuracy interaction analysis in this study.

The time courses generated by dual regression were fed into *FSLNets* (v0.6) to estimate between-network connectivity (Smith et al., 2013). *FSLNets* was run using aggressive component denoising and a full correlation. *FSLNets* uses network modeling in which each variable (in our case, each ICA component) is a node in the model and each connection between any two nodes is an edge. FSLNets produces a node × node matrix of correlation coefficients that represent the strength of connections (edge strength) between any two selected nodes. The effect of the diagnostic status-by-memory accuracy interaction on edge strength was tested using *randomize* with 5000 permutations and TFCE to correct for multiple comparisons corrected-*p* < 0.05. To correct for the number of estimated contrasts (*n* = 2), we used a Bonferroni corrected *p* < 0.025.

##### Exploratory Analysis

Because the effects identified in the analyses described above could be related to severity of current or lifetime depression symptoms as well as the use of medications, we conducted exploratory analyses to examine the associations between severity of current (HDRS-25) and lifetime depression (MOODS-SR depression scale), as well as the age of depressive disorder onset, illness duration, current mood episode duration, and a total medication load with all significant effects identified in the resting state analyses. The exploratory analyses were conducted only in the DD group.

To further examine the heterogeneity of our patient population, we compared clinical, behavioral, and neuroimaging characteristics in individuals with DD who are diagnosed with MDD vs. those diagnosed with PDD, as well as in individuals with DD with and without comorbid diagnoses.

Finally, since we administered the forced-choice recognition test for faces prior to the recognition test for objects, we conducted an exploratory analysis to examine the relationship between accuracy on the facial recognition task with accuracy on the object recognition task and on the overall functional connectivity model.

## Results

### Demographic and Clinical Data Analysis

The two groups did not significantly differ in age, sex, or IQ (Table 1). Individuals with DD, compared to HC, had significantly higher scores of current depression severity measured by HDRS-25, lifetime depression measured by MOODS-SR, and current mania symptoms measured by YMRS (*p* < 0.001).

### Memory Retrieval Analysis

The two groups did not significantly differ in recognition accuracy [HC = 76.3 (1.8); UD 76.9 (1.9), *t*(95) = −0.22, *p* = 0.8] and recognition RT [HC = 968 (25) msec; UD = 1010 (21) msec, *t*(95) = −1.36, *p* = 0.18].

### Resting State Data Analysis

**FIGURE 3:**
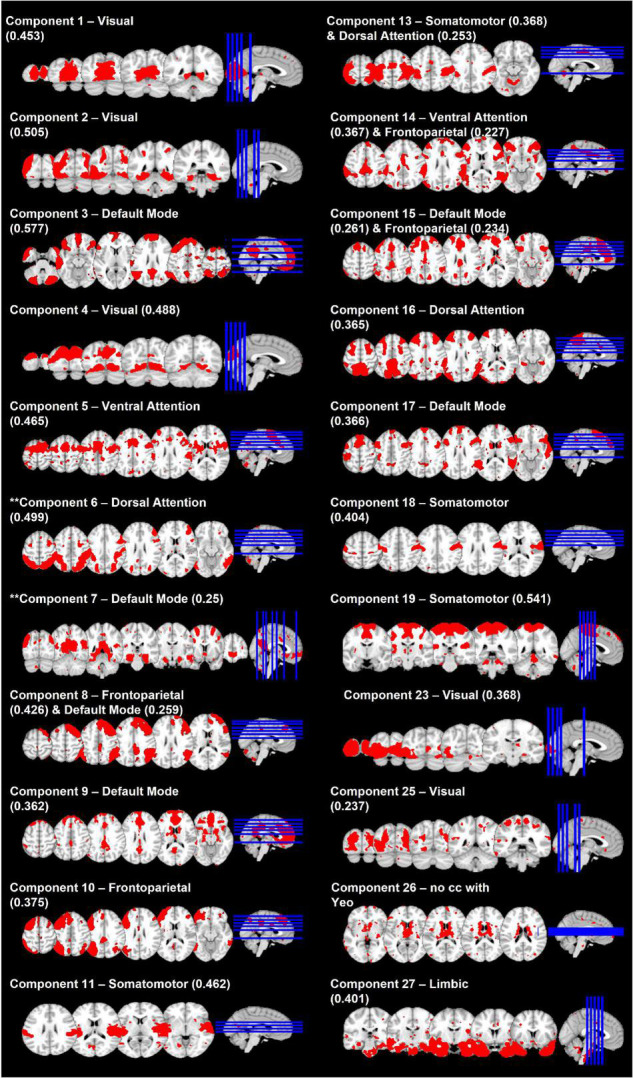
Spatial maps of 22 selected network components. Network labels are based on cross-correlation with Yeo et al. (2011) and values in parentheses indicate the cross-correlation coefficient. ** denotes the two components whose connectivity was related to diagnostic status-by-memory accuracy interaction.

Of the 30 independent components, 21 cross-correlated with Yeo’s 7-network solution (Yeo et al., 2011) with at least 0.2 (Figure 3). During further visual examination, we identified a component that was comprised of regions in the basal ganglia. Even though this component did not correlate with any of Yeo’s networks, we retained it for the further analyses. The remaining eight components followed white matter pathways, represented the cerebellum, or followed patterns of physiological noise and, therefore, were excluded from the further analyses.

*Dual regression* did not reveal any significant effects of diagnosis-by-memory accuracy interaction on functional connectivity in any of 22 independent components.

**FIGURE 4:**
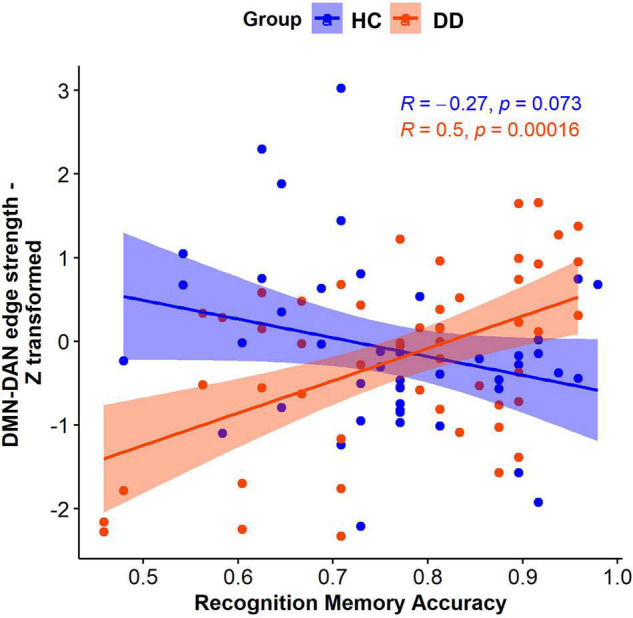
The diagnosis-by-memory accuracy interaction effect on the edge strength between the DMN and DAN in individuals with DD and HC.

*FSLNets* revealed that the strength of connectivity between the independent component (IC) 7 that cross-correlated with the DMN and the IC6 that cross-correlated with the DAN (Figure 4) was significantly associated with the interaction between diagnostic status and memory accuracy (*p* = 0.0156). A *post hoc* analysis showed that this effect was driven by the positive correlation between the connectivity strength and memory accuracy in individuals with DD (*t* = 4.1, *p* = 0.00016). Specifically, the individuals with DD who had more accurate memory for objects had a positive relationship between the DMN and DAN connectivity. However, those with less accurate memory had a negative relationship (anticorrelation) between the DMN and DAN. In HC, the DMN-DAN relationship was not significantly associated with memory accuracy (*t* = −1.8, *p* = 0.07) and, on average, was not different from 0 [one-sample *t*-test: *t*(44) = −0.66, *p* = 0.5].

### Exploratory Analyses

Exploratory analyses investigated whether recognition accuracy and the connection strength between the DMN and DAN were associated with clinical variables in individuals with DD. Neither accuracy nor connection strength between the DMN and DAN were related to current or lifetime depressive symptom severity, age of DD onset, medication load, or duration of current mood episode (all *p*-values > 0.05).

Analyses investigating difference between MDD and PDD revealed that the two groups did not significantly differ in their current or lifetime depressive symptom severity, age of illness onset, or medication load (all *p*-values > 0.05; Table 2). Further, the two disorders did not significantly differ in recognition accuracy [MDD: 77.3 (2.2)%; PDD: 76.2 (13.8)%, *t*(50) = 0.26, *p* = 0.8] and recognition RT [MDD: 1010 (22) msec; PDD: 1019 (48) msec, *t*(50) = −0.2, *p* = 0.84]. Importantly, the linear regression analysis that predicted the DMN-DAN connectivity strength from memory accuracy yielded significant results in the MDD only sample [*F*(1,33) = 4.5, *p* = 0.04], as well as in the PDD only sample [*F*(1,15) = 13.6, *p* = 0.002], which paralleled the findings for the whole DD sample.

**TABLE 2:** Demographic and clinical characteristics of individuals diagnosed with major depressive (MDD) and persistent depressive (PDD) disorders.

	MDD	PDD	Statistics MDD vs. PDD
Number of participants	35	17	
Gender composition (number females)	29	13	chi^2^ = 0.03, *p* = 0.86
Age (years)	27.32 (0.89)	30.02 (2.14)	*t*(50) = −1.38, *p* = 0.18
BMI	25.18 (0.67)	25.4 (0.92)	*t*(50) = −0.19, *p* = 0.85
IQ (NART)	109.12 (1.3)	109.95 (1.55)	*t*(50) = −0.38, *p* = 0.7
Current depression severity (HDRS-25)	11.57 (1.17)	15.35 (1.67)	*t*(50) = −1.85, *p* = 0.07
Current mania severity (YMRS)	1.31 (0.27)	0.88 (0.27)	*t*(50) = 0.99, *p* = 0.33
Lifetime depression (MOODS-SR)	18.51 (0.76)	18.94 (0.81)	*t*(50) = −0.35, *p* = 0.73
Illness Onset (year of age)	14.69 (0.51)	15.35 (1.11)	*t*(50) = −0.63, *p* = 0.53
Number of participants taking Antidepressants	22	7	na
Number of participants taking Mood stabilizers	0	2	na
Number of participants taking Antipsychotics	1	0	na
Number of participants taking Benzodiazepines	4	3	na
Number of participants taking Stimulants	4	0	na
A mean number of psychotropic medications	1.00 (0.14)	1.29 (0.31)	*t*(50) = −1.02, *p* = 0.31
A mean total medication load	1.29 (0.19)	1.65 (0.43)	*t*(50) = −0.9, *p* = 0.37
Number of participants with comorbid diagnoses	20	14	na

Individuals with DD with comorbid disorders had significantly higher lifetime depression severity than those without comorbid disorders based on the MOODS (Dell’Osso et al., 2002) assessment [*t*(50) = 3.3, *p* < 0.005]. Other clinical, behavioral, and connectivity measures were not related to the presence of comorbid disorders.

Finally, although the memory accuracy for faces significantly correlated with memory for objects across all participants (*r* = 0.52, *p* < 0.001), it was significantly lower than the memory accuracy for objects [*t*(96) = −9.6, *p* < 0.001]. Memory accuracy for faces was not associated with the presence of DD diagnosis. Importantly, the connectivity values were not related to the interaction between diagnostic status and memory accuracy for faces [*F*(1,93) = 1.64, *p* = 0.2].

## Discussion

In this study, we investigated the interaction effect of diagnostic status (DD vs. HC) and the strength of memory representations acquired prior to the resting state scan on within- and between-network resting state connectivity. The results indicated that diagnostic status moderated the relationship between memory accuracy and the DMN-DAN connectivity strength. Specifically, the DMN and DAN anticorrelated in the individuals with DD who had lower recognition memory accuracy, but positively correlated in those with DD who had higher recognition memory accuracy. In contrast, the DMN and DAN connectivity was not associated with memory accuracy in HC. We hypothesize that the distinct relationship between memory accuracy and the DMN-DAN connectivity in individuals with DD could indicate aberrant memory reactivation and consolidation processes in depressive disorders because the resting state scan was acquired immediately after the stimulus recognition and was conducted within a timeframe of continued memory consolidation, the results were correlated with memory performance, and one of the networks included bilateral hippocampus.

The DMN increases in activation during rest (Buckner et al., 2008; Smith et al., 2009; Raichle, 2015) and supports perception-independent (Smallwood et al., 2013) and internally oriented cognition, such as autobiographical memory (Andrews-Hanna et al., 2010a), abstract representation of task details in ongoing thought processes (Sormaz et al., 2018), future-oriented thought (Xu et al., 2016), and spontaneous cognition (Buckner et al., 2008; Andrews-Hanna et al., 2010a). In contrast, the DAN increases in activation during cognitive task performance and is implicated in top-down control of attention (Corbetta and Shulman, 2002; Vossel et al., 2014). While the DMN typically anticorrelates with the DAN (Fox et al., 2005; Buckner et al., 2008; Hampson et al., 2010), the degree of such anticorrelation may vary across cognitive states (Dixon et al., 2017) and environmental factors (Qian et al., 2020). For example, one study reported that the regions in these networks co-activated during task preparation but “anti-correlated” during task performance (Koshino et al., 2014). Another study found that the DMN and working memory network (that included the regions comprising the DAN in our study) co-activated during encoding and retrieval phases of a working memory task but anticorrelated during the maintenance phase of this task (Piccoli et al., 2015). Our study contributes to this line of research by showing that the relationship between the DMN and DAN is associated with psychopathology and memory accuracy in the task immediately preceding the resting state scan.

Resting state functional connectivity may reflect experiences acquired prior to the resting state scan (Daselaar et al., 2010; Sami et al., 2014; Cecchetto et al., 2019). Specifically, one study has demonstrated that the patterns of activation observed during encoding can spontaneously reactivate during a subsequent resting state scan (Deuker et al., 2013). Consistent with the memory consolidation theories (McClelland et al., 1995; Squire et al., 2015), consolidation starts in the hippocampus and continues in the neocortex, including the PFC. Given that more accurate memory in individuals with DD, but not HC, was associated with a stronger positive relationship between the DMN (that included the bilateral hippocampus) and DAN (that included the PFC and parietal regions) during the subsequent resting state scan, we hypothesize that these findings indicate aberrant spontaneous memory reactivation and consolidation in these individuals. This pattern of results may indicate that HC disengaged from the task when the task was over, while the individuals with DD who accurately memorized the stimuli in the task were not able to do so. The latter individuals might experience spontaneous reactivation and “replay” of memories even in the absence of direct stimulation. Individuals with more accurate memories might reactivate those memories more than individuals with less accurate memories. Future studies should directly test the spontaneous memory reactivation and consolidation hypothesis because these memory processes could be a neurobiological basis for depressive rumination, which is a repetitive internal thinking pattern focusing on negative experiences and affect (Holtzheimer and Mayberg, 2011). While we did not directly examine memory reactivation at rest or ruminative processes, the previous literature on rumination supports this idea and suggests a relationship between rumination, resting state functional connectivity, and depression. For example, in depressed individuals, this phenomenon was positively associated with functional connectivity in the anterior DMN (Zhu et al., 2012; Lois and Wessa, 2016), negatively associated with functional connectivity in the cortical DMN regions (Rosenbaum et al., 2017), and was associated with more variable functional connectivity between medial prefrontal cortex and insula (Kaiser et al., 2016). Future studies are needed to clarify and test the hypothesized relationship between rumination and memory reactivation and consolidation processes proposed here.

The findings from exploratory analyses in the individuals with DD revealed that the memory accuracy and DMN-DAN correlation were not significantly related to current or lifetime depressive symptom severity, the use of psychiatric medications, age of illness onset, comorbid diagnoses, or diagnosis of MDD vs. PDD in the individuals with DD. While these findings are indicative of general reorganization of connectivity between brain networks in individuals with DD, future studies need to examine other factors affecting the DMN-DAN connectivity in affected individuals.

One limitation of this study is the relatively short duration of the resting state scan. A longer scan time is preferable because it provides more reliable estimates of functional connectivity (Birn et al., 2013). However, longer scan times also increase the likelihood of a participant falling asleep during the scan, which would affect resting state functional connectivity (Soehner et al., 2019). Sleep disturbance is one of the clinical symptoms of DD; therefore, staying awake during a longer resting state scan may be especially challenging for individuals with DD. Another limitation of this study is the lack of a baseline resting state scan performed prior to stimulus encoding or between encoding and memory retrieval. Many studies that examined the relationship between resting state functional connectivity and task performance included an additional resting state scan prior to task performance to capture baseline functional connectivity to better distinguish “trait-like” from “task-related” changes. However, determining the baseline in individuals with mood disorders might be challenging because there is no way to eliminate bias or interference from previous experiences and thus, baseline resting state data may still be affected by rumination or thoughts about previous experiences. Although we hypothesized that our findings might be related to aberrant reactivation and consolidation processes in the individuals with DD, our study was not designed to directly measure consolidation processes. Future studies should modify the existing experimental paradigm by adding a baseline resting state scan between encoding and memory retrieval as well as the second memory test after the resting state scan to test the consolidation hypothesis. Future studies should also incorporate rumination surveys and inquire about the specific thought content that participants experience during rest to discern how ruminative or other thinking patterns are related to basic memory consolidation processes in depression. In this study, we used the pictures of neutral everyday objects. However, the notion of “neutral” stimuli should be interpreted with caution in mood disorders research because affected individuals tend to misinterpret neutral stimuli as emotional (Manelis et al., 2019). Future studies should specifically examine the relationship between resting state functional connectivity and memory for positive vs. negative information immediately preceding the resting state scan.

In summary, we have demonstrated that connectivity strength between the DMN and DAN during resting state was significantly associated with the interaction between participants’ diagnostic status (DD or HC) and memory accuracy in the task preceding the resting state scan. We hypothesize that these differences may relate to aberrant memory consolidation in depression, which may in turn be a basis for depressive rumination and an inability to disengage from thoughts and feelings associated with previous experiences. Understanding the relationship between resting state connectivity and previous experiences may inform the development of interventions strategies alleviating repetitive and intrusive thoughts in depression. However, future studies are needed to clarify the relationship between post-encoding and retrieval DMN-DAN resting state functional connectivity, memory consolidation, and rumination.

## Data Availability Statement

The raw data supporting the conclusions of this article will be made available by the authors, without undue reservation.

## Ethics Statement

This study was approved by the University of Pittsburgh Institutional Review Board. The patients/participants provided their written informed consent to participate in this study.

## Author Contributions

SS acquired the data, evaluated data quality, analyzed and interpreted the data, and drafted and critically evaluated the manuscript. YH curated the data organization and analyses and drafted and critically evaluated the manuscript. RR and ML acquired the data, evaluated data quality, and critically evaluated the manuscript. MP curated the study development, interpreted the data, and critically evaluated the manuscript. HS curated the study development, participants’ recruitment, and evaluation, and critically evaluated the manuscript. AM obtained funding, designed the study, acquired the data, evaluated data quality, analyzed and interpreted the data, and drafted and critically evaluated the manuscript. All authors have read and approved the final version of the manuscript and agreed to be accountable for all aspects of this work.

## Conflict of Interest

HS receives royalties from Wolters Kluwer, royalties and an editorial stipend from APA Press, and served as a consultant for Intracellular Therapeutics and Medscape/WebMD. The remaining authors declare that the research was conducted in the absence of any commercial or financial relationships that could be construed as a potential conflict of interest.

## Publisher’s Note

All claims expressed in this article are solely those of the authors and do not necessarily represent those of their affiliated organizations, or those of the publisher, the editors and the reviewers. Any product that may be evaluated in this article, or claim that may be made by its manufacturer, is not guaranteed or endorsed by the publisher.
